# Topological Nanophotonic Wavelength Router Based on Topology Optimization

**DOI:** 10.3390/mi12121506

**Published:** 2021-11-30

**Authors:** Hongyi Yuan, Zhouhui Liu, Maoliang Wei, Hongtao Lin, Xiaoyong Hu, Cuicui Lu

**Affiliations:** 1 Key Laboratory of Advanced Optoelectronic Quantum Architecture and Measurements of Ministry of Education, Beijing Key Laboratory of Nanophotonics and Ultrafine Optoelectronic Systems, School of Physics, Beijing Institute of Technology, Beijing 100081, China; hy_yuanbit@qq.com (H.Y.); 398812028@qq.com (Z.L.); 2 State Key Laboratory of Modern Optical Instrumentation, College of Information Science and Electronic Engineering, Zhejiang University, Hangzhou 310027, China; ml_wei@zju.edu.cn; 3 State Key Laboratory for Mesoscopic Physics, Department of Physics, Collaborative Innovation Center of Quantum Matter & Frontiers Science Center for Nano-Optoelectronics, Beijing Academy of Quantum Information Sciences, Peking University, Beijing 100081, China; 4 Collaborative Innovation Center of Light Manipulations and Applications, Shandong Normal University, Jinan 250358, China

**Keywords:** on-chip integration, topological photonic crystal, wavelength router, topology optimization

## Abstract

The topological nanophotonic wavelength router, which can steer light with different wavelength signals into different topological channels, plays a key role in optical information processing. However, no effective method has been found to realize such a topological nanophotonic device. Here, an on-chip topological nanophotonic wavelength router working in an optical telecom band is designed based on a topology optimization algorithm and experimentally demonstrated. Valley photonic crystal is used to provide a topological state in the optical telecom band. The measured topological wavelength router has narrow signal peaks and is easy for integration. This work offers an efficient scheme for the realization of topological devices and lays a foundation for the future application of topological photonics.

## 1. Introduction

With the rapid development of integrated nanophotonic technology, the demand for high-performance nanophotonic devices with a small footprint and different functionality is increasing. Among these nanophotonic devices, wavelength router which can steer light with different wavelengths to different channels has broad application in many areas and plays a key role in integrated nanophotonic chips [1,2,3]. Topological photonics, with features of robustness and topological protection, provides a novel platform for topological devices [4,5,6]. For example, topological wavelength router can guide light with different wavelengths to different topological channels, which can increase the robustness of integrated nanophotonic chips and has good potential in the application of on-chip light signal processing in the future.

There are various schemes to achieve topological photonics, including gyromagnetic photonic crystals [7,8], bi-anisotropic metamaterials [9], and dielectric material [10]. Among them, valley photonic crystal (VPC) [11,12], as an important kind of topological photonic system, has great application potential in on-chip nanophotonic devices because it can work in optical telecom band and is convenient for on-chip integration. Instead of breaking time reversal symmetry, the reduction of space inversion symmetry leads to nonvanishing valley-dependent Berry curvature and quantum valley Hall effect in VPC [13]. Due to the reservation of time reversal symmetry, VPC is free of external magnetic field, which is beneficial for on-chip integration in optical frequency range. The simple structure is also friendly to current micro-nano fabrication. These facts make VPC convenient for experiments and give a fascinating prospect in designing on-chip integrated high-performance nanophotonic devices [14,15,16,17]. Works of devices designed with VPC are presented in recent years, including topological Mach–Zehnder interferometers [18], topological photonic routing structure [11,19], valley beam filter [20], photonic detouring structure [21], and splitters [22]. However, much attention has been paid to engineer the bulk band dispersion to realize topological states in the realm of topological photonics, and a topological wavelength router working in optical telecom band has not been reported to date, which has restricted the practical application of topological states and the development of robust nanophotonic devices.

In this work, a topological nanophotonic wavelength router working in optical telecom wavelength range is realized based on topology optimization for the first time. The designed nanostructures are fabricated and measured successfully. The topological wavelength router works in the bandgap of the designed VPC and can separate and steer different incident optical wavelengths into different output ports. The full widths of the half maximum of the transmission peaks are 5 nm for 1520 nm and 6 nm for 1550 nm. The signal-to-noise ratio of two peaks are 11.20 dB and 15.76 dB, respectively. By introducing topological waveguides, the wavelength router can achieve narrow peaks and low cross talks at the same time. The experimental results show that the designed wavelength router is easy to be fabricated and convenient to be integrated. Furthermore, some external perturbations are introduced into the device to prove the robustness of the device. The topological nanophotonic wavelength router based on topology optimization offers an efficient scheme in the field of topological devices and paves the way for the application of topology photonics in high-performance nanophotonic devices.

## 2. Device Structure and Optimization Methods

**Figure 1:**
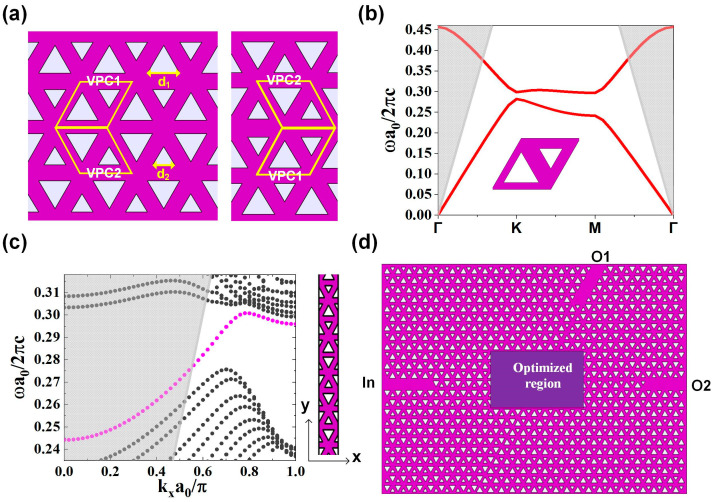
(**a**) The structure of VPC. The unit cell, with lattice constant a = 445 nm, consists of two equilateral triangular holes of side lengths d_1_ = 0.6 a and d_2_ = 0.4 a. The input and output channels of wavelength router are designed with the interfaces of two lattices, VPC1 and VPC2. (**b**) Band structure of the unit cell. Γ, K and M denote the high-symmetry points in the momentum space. The frequencies above the light cone is shaded with gray color. (**c**) Topological edge state. The corresponding structure is shown on the right, which is periodic along the x direction and finite along y direction. It can be seen that the edge state (magenta) crosses the bandgap between the bulk bands (gray). The frequencies above the light cone is shaded with gray color. (**d**) Structure diagram of optimized topological wavelength router. The input port “In” is on the left side. “O1” and “O2” are the upper output port and the right output port, respectively. The rectangle in the middle is the area to be optimized.

In the beginning, a valley Hall photonic crystal with the properties of topological protection is designed in order to provide a platform for topological photonic state at telecom wavelengths. The structure of the designed VPC is shown in Figure 1a. The unit cell with a lattice constant of a = 445 nm consists of equilateral triangular holes of side lengths d_1_ = 0.6 a and d_2_ = 0.4 a. The space inversion symmetry is broken by setting two equilateral triangular holes with different side lengths. The input and output channels consist of the interfaces of two lattices, VPC1 and VPC2, as shown in Figure 1a.

To demonstrate the topology property of the structure, dispersion band of the unit cell and edge state are calculated. From Figure 1b, it can be seen that the Dirac point at K point is open and there is a valley on each side of the band gap. The band gap ranges from 0.28186 (1490.6 nm) to 0.29854 (1578.8 nm). The band structure of the edge state is shown in Figure 1c. The corresponding geometry is shown in the inset on the right, which is set to be periodic along x direction and finite along y direction (five unit cells in each region). From the band diagram, it can be seen that the edge state (magenta) crosses the band gap of bulk states (gray). It should be pointed out that the valley dependent edge state has different origin from those in photonic quantum Hall systems by breaking time reversal symmetry, which is the reason why the edge state doesn’t connect upper and lower bulk bands directly [23]

The structure diagram of the optimized topological router is shown in Figure 1d. The input port (“In”) is on the left interface boundary of two lattices VPC1 and VPC2 while the output ports are on upper interfaces boundary (“O1”) and righter interfaces boundary (“O2”). Photonic crystal waveguides with a missing row of triangular air holes below the interface are used to further improve the light injection performance, which can significantly decrease the scattering losses at the input and output interfaces [24]. Several periods of lattice are removed from the center of the structure, and the center region is to be optimized by the algorithm to realize the topological wavelength router. The functionality of the central area is to guide signals with different wavelengths to different topological channels.

**Figure 2:**
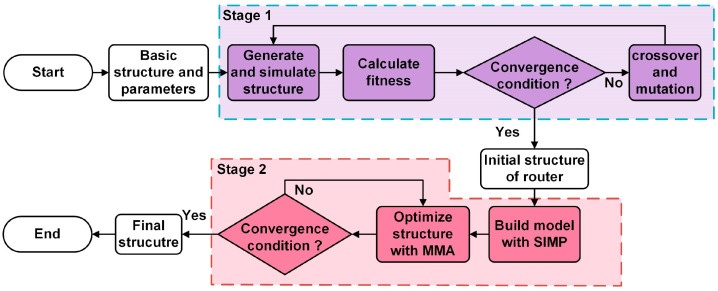
Schematic diagram of optimization method. Stage 1 is the design process of initial structure which is optimized with topology optimization in stage 2.

The optimization aim of the algorithm is to find the best distribution of two kinds of material, which can fulfill the design target under given conditions. The key point of optimization is to express the target correctly with formulas, where the target is expressed with transmission of each channels. The optimization process is shown in Figure 2, which has two main stages. The first stage is the generation of the initial structure. The second stage is topology optimization. Topology optimization, as an important kind of optimization method, is at first applied in mechanics [25] and has been broadly used in many areas including nanophotonic design in recent years [26,27,28,29].

The significance of topology optimization is obvious in our work because it enlarges the parameter space greatly to achieve freeform structure design and can realize high performance under given conditions. From the perspective of mathematics, topology optimization method is essentially based on gradient descent, which means topology optimization is sensitive to initial geometry. It is shown that randomly generated initial geometry can give unexpected high performance after topology optimization [30]. Noticing the randomness during the optimization process of genetic algorithm, a new optimization method is established, which employs genetic algorithm to generate initial geometry of topology optimization. In this way, the whole optimization process can be divided into two stages: the stage of generation of initial structure and the stage of topology optimization.

In the first stage, random numbers are used to represent the structure. First, initial conditions and basic structure of optimization are set, such as materials, area size and target wavelength. The center position, width and height of cells are taken as random variables. Second, random numbers are used to generate a series of variables that correspond to a series of geometric structures, and the structures are simulated by using finite element method (FEM). Third, the electromagnetic field response solved by FEM is used to evaluate the individual’s fitness. The transmittance on the objective output port is regarded as signal, while the transmittance on the other output port is regarded as noise. The difference between the noise and signal is the fitness to assess each structure [31,32]. Fourth, crossover and mutation are used to generate new structures. After several repeated iterations of the first loop shown in Figure 2, the initial structure of router optimized in the first stage is sent to topology optimization. In addition to FEM, another common numerical calculation method finite-difference time-domain (FDTD) method can also be adopted to combine with topology optimization to design topological nanophotonic router devices.

In the second stage of topology optimization, the initial structure is modeled by variable density method, i.e., Solid Isotropic Material with Penalization (SIMP). SIMP makes interpolation between the indexes of two materials and introduces penalization function into optimization to converge to binary structure [33,34]. SIMP deals with the density of each point, which means variable number is proportional to grid density. The variable number is extremely large and Method of Moving Asymptotes (MMA) is used to optimize the problem [35]. After iterations of the second loop shown in Figure 2, a topological router that reaches convergence conditions will be obtained at the end of the second stage. Compared with traditional design method like free propagation region in arrayed waveguide gratings, our method can achieve wanted function with a much smaller footprint, which is good for on-chip integration.

## 3. Optimization Results and Experimental Verification

The results of the topological router designed by the method above is presented below. The operation range of the router is set within the band gap, which is from 1500 nm to 1570 nm. The target wavelengths are 1520 nm for port O1 and 1550 nm for port O2.

**Figure 3:**
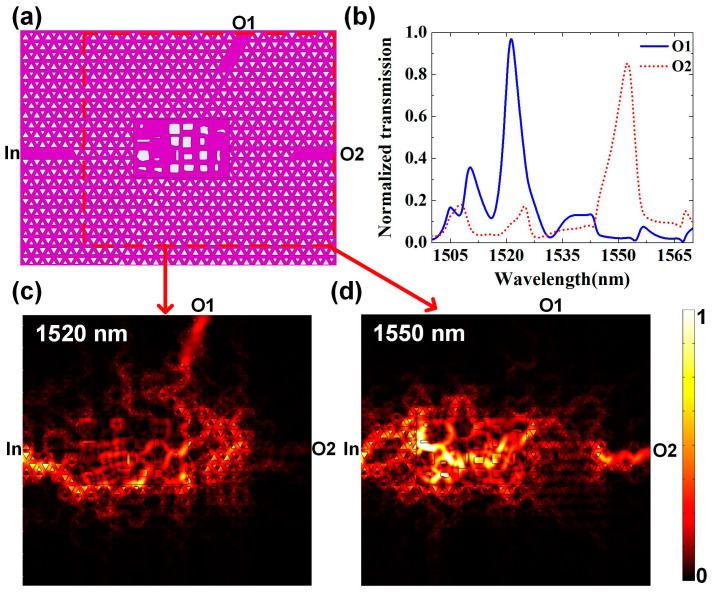
Calculated result of topological wavelength router. (**a**) Structure diagram of the topological wavelength router. The purple area represents silicon and the white area represents air. The size of internal optimization area is 2.30 μm × 3.78 μm. (**b**) The simulated transmission spectrum covers from 1500 nm to 1570 nm, which is within the band gap of designed VPC shown in Figure 1. The central wavelengths are 1520 nm and 1550 nm corresponding to the transmission spectrum peaks of the upper (“O1”) and right (“O2”) channels, respectively. (**c**,**d**) The simulated time-average power flow distribution at 1520 nm and 1550 nm near the central optimized area, respectively. The potting area corresponds to the red rectangle marked in (**a**).

The final optimized structure is shown in Figure 3a. The purple area represents silicon and the white area represents air. The size of internal optimization area is 2.30 μm × 3.78 μm. The simulated transmission spectrum of the topological router is shown in Figure 3b, which covers from 1500 nm to 1570 nm. The central wavelengths are 1520 nm and 1550 nm corresponding to the transmission spectrum peaks of the upper (“O1”) and right (“O2”) channels, respectively. In the first stage of the optimization process, 24 randomly generated rectangles are set as inner geometries and the population size is set as 20. The side length of each rectangle is constrained no less than 50 nm, which takes fabrication accuracy into consideration. The first stage converges after 150 times of iteration. In the second stage, the max simulation number is set as 100 and the second stage converges when the number of simulations exceeds this value. The time cost is about 5 h for the first stage and about 21 h for the second stage. The whole optimization process is calculated on a server loaded with an Intel(R) Xeon(R) CPU E5-2690 v4 @ 2.60GHz.

It can be seen that the crosstalk is low and the bandwidth of each peak is narrow, which largely depends on the introduction of topological waveguides. The signal-to-noise ratio of two peaks are 11.20 dB and 15.76 dB, respectively. The full width of the half maximum of the transmission spectrum of the two output ports is only 5 nm for 1520 nm and 6 nm for 1550 nm. Figure 3c,d show the simulated time-average power flow distribution at 1520 nm and 1550 nm. It should be pointed out that Figure 3c,d only show the field distribution in the optimized area and the part of two output channels, for the purpose of clearness. From these two figures, it can be seen that the field is a mazed area with topological boundaries. It can also be seen that the upper left corner and lower right corner can be cut off to further decrease the device footprint. From Figure 3c,d, it can be seen that the field density is bigger than in two output channels, which can be explained with the backscattering caused by central optimized structure. The backscattered energy at the interface between the VPC waveguide and the optimized area is calculated. When light ranging from 1500 nm to 1570 nm is injected from the left port, with power intensity of 1 W/m, the average backscattered energy intensity is 0.129 W/m. The backscattering can be further decreased by taking the structure near the interface into consideration in the optimization. There is also some weak energy leaking into the lower VPC boundary, which can be used to extend our scheme to more channels and narrower wavelength spacing.

**Figure 4:**
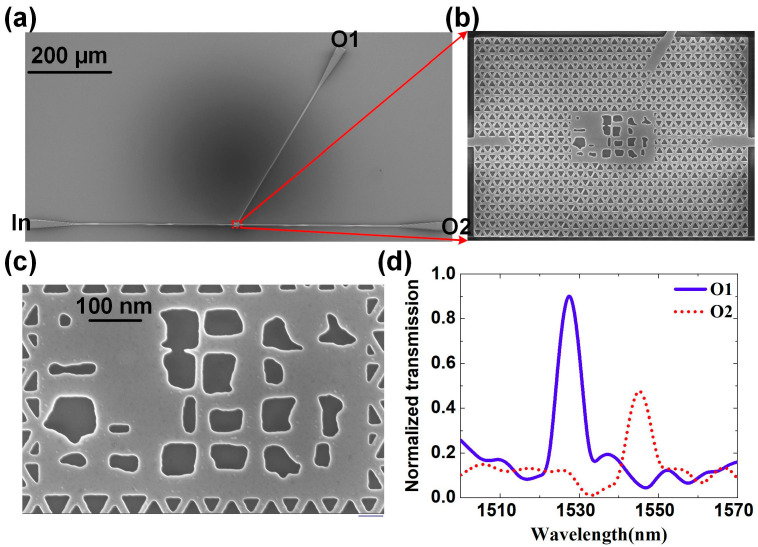
Experimental fabrication and measurement of a two-channel topology router. (**a**) SEM top view of the whole etched sample. (**b**) The enlarged SEM image of topological router components. (**c**) The SEM image of the central optimized structure. (**d**) The normalized transmission spectra measured experimentally.

The designed topological nanophotonic router is fabricated and measured experimentally. The sample was fabricated on silicon-on-insulator (SOI) platform with a 220 nm device layer on top of a 2-μm SiO_2_ BOX layer. Firstly, the SOI were cleaned with O_2_ plasma (PT-5SM). A layer of positive-tone resist (AR-P 6200.13) was spin-coated. The waveguides were patterned by electron-beam lithography (EBL, Raith Voyager) and subsequently etched using inductively coupled plasma (ICP, SAMCO RIE-101iPH) with the gases of SF6, C4F8, and Ar. Secondly, the valley photonic crystal structures were fabricated through the same process. Finally, the grating couplers were patterned and etched to the depth of 70 nm. The scanning electron microscopy (SEM) image of the etched sample is shown in Figure 4a. Figure 4b shows the enlarged SEM image of the topology router, and the SEM image of the center designed structure is shown in Figure 4c, which is designed based on topology optimization with size of 2.30 μm × 3.78 μm. It can be seen that the etched structure is basically the same as the design.

An optical fiber-coupled microscope measurement system was used to measure the performance of the sample, which is the same measurement method as stated in our previous works [31,32], and the normalized transmission spectrum obtained by the test is shown in Figure 4d. The normalized transmission is defined by comparing the measured light intensity of etched sample to the one of an unetched sample. There are obvious transmission peaks around 1520 nm and 1550 nm, and the bandwidth is very narrow. Due to the influence of etching technology and experimental test environment, the transmission around 1550 nm is lower than the simulated one and the central wavelengths of signals are slightly different from targets. It is possible to improve the measured performance by increasing the signal intensity, reducing environmental noise, improving fabrication technology, and improving test means. It is necessary to point out that there are inevitable deviations in the fabricated samples from ideal design, because our EBL’s alignment accuracy is 100 nm. However, the test results show that tiny deviations have little influence on the device’s functionality.

## 4. Discussion

**Figure 5:**
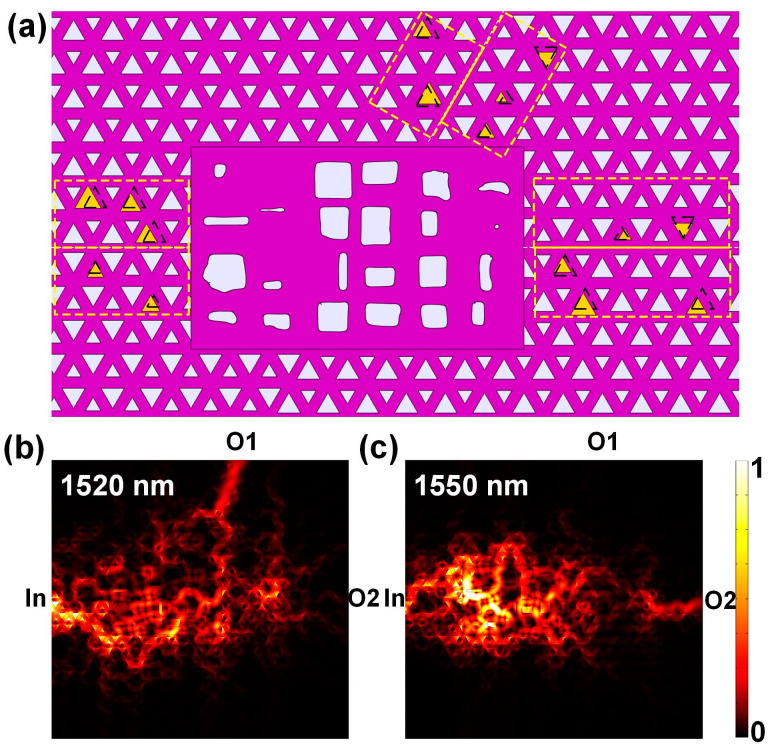
Calculated results of structure after introducing disorders. (**a**) Schematic diagram of the structure with disorders introduced. The randomly adjusted triangular holes are colored with yellow. The dotted triangles show the original shape and position of the adjusted triangles. (**b**,**c**) Calculated time-average power flow distribution at 1520 nm and 1550 nm near the central optimized area, respectively.

In order to prove the robustness of the designed wavelength router, some random disorders are introduced into the device. The way of introducing disorders is randomly adjusting the triangles around three channels, which is shown in Figure 5a. Triangles in the yellow dotted boxes are randomly chosen and adjusted. Yellow triangles are the triangles being adjusted. For simplicity, only the size and position are taken into consideration. The side length of one randomly adjusted triangle ranges from 0.3 a to 0.7 a. The direction of the movement is chosen randomly and the length of the movement ranges from 0.052 a to 0.144 a.

By introducing disorders in the device, the periodic property of the lattice is broken. However, the calculated results show that the designed device can still work as expected. Figure 5b,c show the simulated time-average power flow distribution at 1520 nm and 1550 nm near the central optimized area, respectively. From the field distribution figures, it is clear to see that the external perturbation has little influence on the functionality of the device.

## 5. Conclusions

In conclusion, an on-chip topological wavelength nanophotonic router based on topology optimization in the telecom wavelength range is designed and experimentally realized for the first time, which steers signals with different wavelength into different ports. The topological wavelength router works in the bandgap of the designed valley photonic crystal and possesses the characteristics of narrow peaks and easy integration. The full widths of the half maximum of two peaks are 5 nm for 1520 nm and 6 nm for 1550 nm, respectively. Besides, external perturbations are also introduced into the device to demonstrate the robustness. The topology-optimized topological router based on valley Hall photonic crystal offers an efficient scheme in the field of topology nanophotonic devices and paves the way for the application of topology photonics in high-performance nanophotonic devices.
